# Bromination and increasing the molecular conjugation length of the non-fullerene small-molecule acceptor based on benzotriazole for efficient organic photovoltaics†

**DOI:** 10.1039/d1ra01348c

**Published:** 2021-04-13

**Authors:** Na Zhang, Zhe Li, Can Zhu, Hongjian Peng, Yingping Zou

**Affiliations:** College of Chemistry and Chemical Engineering, Central South University Changsha Hunan 410083 China hongjianpeng@126.com +86-731-88879616

## Abstract

Two novel non-fullerene acceptors, namely BZIC-2Br and Y9-2Br, were synthesized by employing a ladder-type electron-deficient-based fused ring central with a benzotriazole core. Y9-2Br is obtained by extending the conjugate length of BZIC-2Br. Compared with BZIC-2Br, Y9-2Br possesses a lower optical bandgap of 1.32 eV with an absorption edge of 937 nm, exhibiting broader and stronger absorption band from 600 to 900 nm. Moreover, Y9-2Br exhibits excellent photovoltaic properties with *V*_oc_ of 0.84 V, *J*_sc_ of 21.38 mA cm^−2^ and FF of 67.11%, which achieves an impressive PCE of 12.05%. Our study demonstrates that bromination and effective extension of the conjugate length can modulate performance from different aspects to optimize photovoltaic characteristics.

## Abstract 2

Two novel non-fullerene acceptors, namely BZIC-2Br and Y9-2Br, were synthesized by employing a ladder-type electron-deficient-based fused ring central with a benzotriazole core.

## Graphical abstract

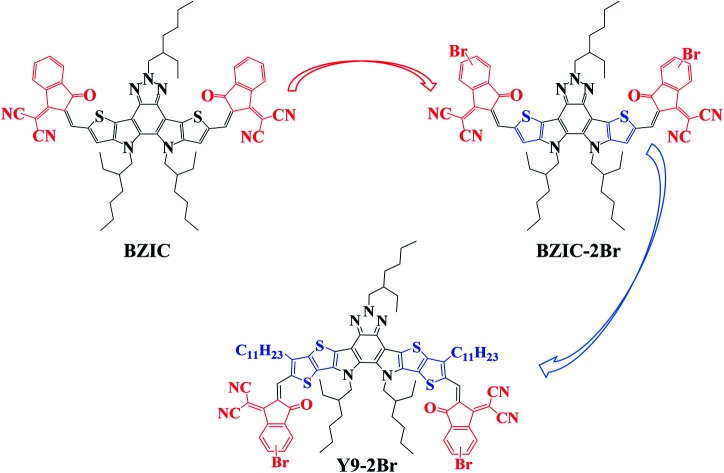

## 1. Introduction

**Fig. 1:**
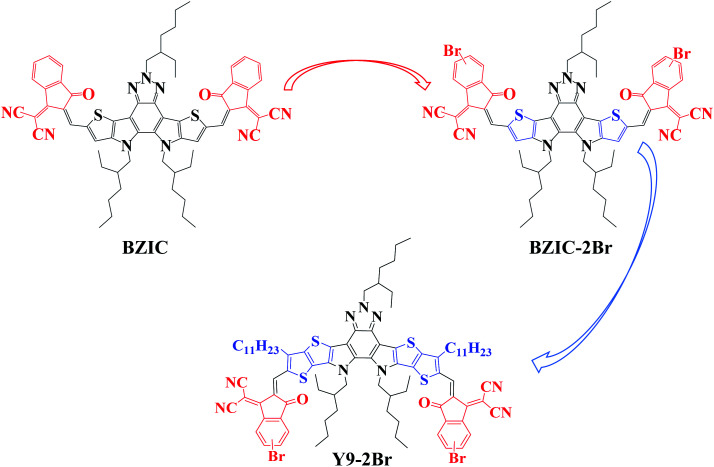
The chemical structures of BZIC, BZIC-2Br and Y9-2Br.

The photoactive layers of bulk-heterojunction (BHJ) organic solar cells (OSCs) are usually constructed with electron donors and acceptors.^1^ Electron acceptors include commonly fullerene-based acceptors or non-fullerene fused-ring acceptors (NFAs). Over the past two decades, great efforts have been made to improve the power conversion efficiency (PCE) by over 18% for single-junction devices.^2–5^ In general, the performance of OSCs can be evaluated by the following three main parameters: the open circuit voltage (*V*_oc_), short-circuit current density (*J*_sc_) and fill factor (FF). Among acceptor innovations, NFAs have become the main materials of OSCs because of reduced energy loss for a high *V*_oc_ and extended absorption range for a high *J*_sc_, which is essential requirements for high PCE.^6,7^ The first breakthrough was made in 2015 for the development of NFAs. Zhan *et al.* reported a fused-ring electron acceptor (FREA), named ITIC, with an acceptor–donor–acceptor (A–D–A) structure.^3^ The structure was further optimized to boost the PCE of OSCs to 13–15%.^8–12^ In order to further improve the photoelectric properties of A–D–A type NFAs, an electron-deficient (A′) unit was introduced into the central fused-ring of the A–D–A molecule to form an A–DA′D–A structure by Zou *et al.*, named BZIC^2^ (Fig. 1), based on a ladder-type thieno[3,2-*b*]pyrrolo-fused pentacyclic benzotriazole (BZTP) as the core and end-capped with 1,1-dicyanomethylene-3-indanone (INCN),^13–15^ which exhibits an excellent PCE of 6.3% with *V*_oc_ of 0.84 V, *J*_sc_ of 12.67 mA cm^−2^ and FF of 59%. The emergence of Y6 in 2019 broke the record for the highest OSCs at that time, with PCE exceeding 15%. On this basis, the subsequent development of Y series NFAs is constantly updated, promoting the progresses of OSCs with the high PCE ranging of 15–18%.^5,16,17^ Compared to ITIC-series FREAs, the main difference of Y-series NFAs is the introduction of the electron-deficient core. Besides, the pyrrole ring is also used to replace cyclopentadiene.^1,18^ The A–DA′D–A-type molecules possess typical structural superiority. First, in order to strengthen D–A interactions and enhance intermolecular and intramolecular interactions, the electron-deficient unit, such as benzothiadiazole (BT), was introduced^19^ into the fused ring, contributing to improve the electron mobility and molecular aggregation.^20^ For the conjugated system, pyrrole-bridging rings can be used as electron donors due to their strong electron-donating ability,^21^ which is beneficial to upshift the highest occupied molecular orbital (HOMO) energy level and reduce the bandgap of NFAs. Finally, there are two nitrogen atoms in the two pyrrole units, which link to the alkyl chains^22^ to suppress severe aggregation due to their steric hindrance and improve the solubility of NFAs. The typical A–DA′D–A structure makes for a low nonradiative recombination loss from electroluminescence quantum efficiency based on these acceptors.^23,24^

Therefore, the structure optimization of Y-series NFAs generally starts from the following three aspects: (1) core engineering influencing the energy levels to regulate the performance of devices.^25,26^ For example, Y1, with benzotriazole (BT) as an electron-deficient core, showed the strong absorption with a narrow bandgap of 1.44 eV.^27^ To improve the photovoltaic performance further, BT was replaced by benzothiadiazole (BTA) to obtain Y5, which showed the deeper HOMO level and the narrower bandgap of 1.38 eV, when compared with Y1;^28^ (2) alkyl chain engineering to improve the solubility of materials and induce steric hindrance effects to minimize the energetic disorder.^16,24,29^ For instance, Hou *et al.* designed a novel molecule named BTP-4F-12, which was obtained by changing 2-ethylhexyl on Y6 to 2-butyloctyl. BTP-4F-12 not only showed better solubility but also tighter π–π stacking, which resulted in an improved PCE of 16.4%, which is superior to that of Y6 (15.7%);^30^ (3) terminal engineering can be used to adjust energy levels, absorption, blend morphology and charge dynamics.^31^ Chen *et al.* synthesized an NFA, BTP-M, by one methyl group instead of fluorine atoms on the IC terminal, showing higher energy levels and blending with PBDB-T, OSCs with a PCE of 14.24%.^32^

Halogens have been widely used in organic semiconductors.^33–35^ The introduction of halogen atoms can adjust the photoelectric properties of materials, such as the absorption and energy levels.^36–42^ Therefore, for non-fullerene OSCs, halogenation is a very promising molecular design strategy to further improve the performance.^43^ Compared to the fluorination and chlorination, the bromine atom is rarely incorporated into the non-fullerene acceptors. In 2017, Li *et al.* explored the bromination of NFAs^44^ and then Chen *et al.* found that bromine atoms were introduced into NFAs, showing the same or even better device performance in comparison with F and Cl.^45^ Zhang further studied the effects of introducing one or two bromine atoms into NFAs, and the results show that one bromine-substituted molecule exhibits better device performance.^46^ Therefore, when the brominated small molecules are used as acceptors, the device performances may be further improved.^47–50^ In addition, increasing the molecular conjugation length of molecules is another measure to optimize photovoltaic properties.^51–56^ The first reported A–DA′D–A NFA, BZIC, showed a moderate PCE of 6.3%.^2^ When the pentacyclic ring was extended to a heptacyclic one to obtain Y1, resulting in PCEs of 13.42%, the reason of which is that increasing the molecular conjugation length can improve light absorption capability and charge transport.^28^

Therefore, based on the above-mentioned strategies, BZIC-2Br (bromination of BZIC) and Y9-2Br (increasing the molecular conjugation length of BZIC-2Br) were designed and synthesized, respectively (Fig. 1). With the introduction of bromine atom, the absorption spectra of BZIC-2Br and Y9-2Br exhibit red-shift to obtain a narrower optical bandgap. Increasing the molecular conjugation length of molecules can regulate the HOMO and the lowest unoccupied molecular orbital (LUMO) energy levels, enhance the light-harvesting capability of molecules, broaden the absorption range, reduce the bandgap and enhance charge mobility. Finally, Y9-2Br exhibits the best excellent performance compared with BZIC and BZIC-2Br.

## 2. Results and discussion

### 2.1 Synthesis and characterization

As shown in Scheme S1 (ESI),† BZIC-2Br and Y9-2Br were synthesized *via* Knoevenagel condensation reactions between the corresponding dialdehyde and end group (2-(6-bromo-3-oxo-2,3-dihydro-1*H*-inden-1-ylidene)malononitrile (INCN-Br)), respectively. The chemical structures of these new small molecule acceptors (SMAs) were fully characterized by ^1^H nuclear magnetic resonance (NMR). The molecular mass was confirmed using an Autoflex III matrix-assisted laser desorption ionization mass spectrometer (MALDI-TOF-MS) (Fig. S11†). Thermogravimetric analysis (TGA) was conducted at a heating rate of 10 °C min^−1^ under nitrogen, as shown in Fig. S12,† which indicates that BZIC-2Br and Y9-2Br have good thermal stability with decomposition temperatures at 310 °C and 318 °C, meeting the requirements of device fabrication. BZIC-2Br and Y9-2Br show good solubility in commonly used solvents, including chloroform (CF) and dichloromethane (DCM), particularly greater than 20 mg mL^−1^ in chloroform at room temperature.

### 2.2 Photoelectric properties

#### 2.2.1 Electrochemical properties

**Fig. 2:**
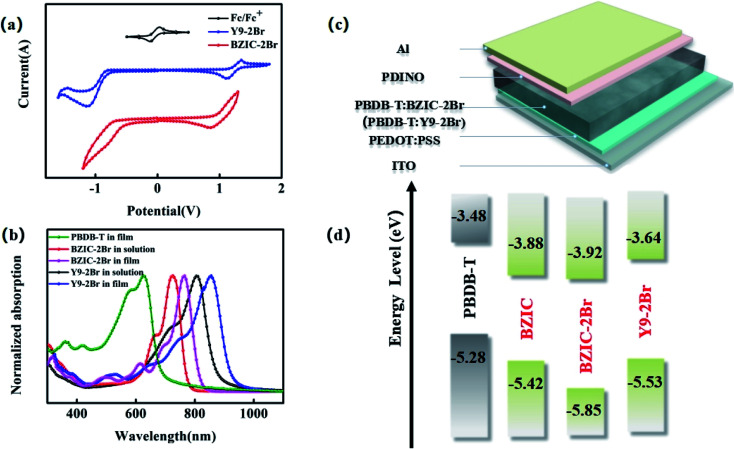
(a) Cyclic voltammetry curves of BZIC-2Br and Y9-2Br; (b) UV-Visible absorption spectra of PBDB-T in film, BZIC-2Br and Y9-2Br in solution and thin film state, respectively; (c) device structure used for OSC fabrication; (d) energy level diagrams of donors (PBDB-T), and acceptors (BZIC,^2^ BZIC-2Br and Y9-2Br).

The HOMO energy level and LUMO energy level of BZIC,^2^ BZIC-2Br and Y9-2Br were measured *via* the cyclic voltammetry (CV) method. As shown in Fig. 2(a), the initial reduction/oxidation potentials of BZIC, BZIC-2Br and Y9-2Br are −0.48/1.06, −0.51/1.45 and −0.73/1.17 V, respectively. Thus, according to the equation *E*_HOMO_/*E*_LUMO_ = −*e*(*φ*_ox_/*φ*_red_ + 4.41), the HOMO/LUMO energy levels of BZIC, BZIC-2Br and Y9-2Br are −5.42/−3.88, −5.85/−3.92, −5.53/−3.64 eV, respectively. In addition, the electrochemical bandgaps are 1.54 eV, 1.93 eV and 1.89 eV (*E*^CV^_g_ = *E*_LUMO_ − *E*_HOMO_), respectively. Compared with BZIC, the LUMO level of BZIC-2Br shows a downward trend, resulting from the electron-withdrawing effects of bromine atoms.^57^ And thieno[3,2-*b*]thiophene instead of a thiophene segment of the molecules can extend the fused central ring, which elevates the LUMO energy levels. The higher LUMO energy level of the acceptor is beneficial for the OSC device to obtain a higher *V*_oc_.

#### 2.2.2 Optical properties

Fig. 2(b) shows the ultraviolet visible (UV-Vis) absorption spectra of the small molecule acceptors BZIC-2Br and Y9-2Br in the chloroform solution and thin film. The relevant optical data are listed in the Table 1. From the spectra, it can be seen that BZIC-2Br and Y9-2Br have absorptions in a range of 350–940 nm. In particular, the maximum absorption peaks are 807 nm and 855 nm in the film, and the edges of absorption are 886 nm and 937 nm in the film. Compared with BZIC, BZIC-2Br and Y9-2Br exhibit a significant red-shift, as shown in the absorption spectrum (about 29 and 80 nm) and the narrower optical bandgap (*E*^opt^_g_, *E*^opt^_g_ = 1240/*λ*_edge_) of 1.40 and 1.32 eV in the thin film, which shows that bromination and increasing the molecular conjugation length can enhance the light-harvesting capability and reduce the bandgap.

**Table 1:** Photoelectric data of BZIC-2Br and Y9-2Br in the film state

Parameter	*λ*_max_ (nm)	*λ*_edge_ (nm)	*E* ^opt^_g_ (eV)	*E*_HOMO_ (eV)	*E*_LUMO_ (eV)	*E* ^CV^_g_ (eV)
BZIC^2^	770	857	1.45	−5.42	−3.88	1.54
BZIC-2Br	807	886	1.40	−5.85	−3.92	1.93
Y9-2Br	855	937	1.32	−5.53	−3.64	1.89

### 2.3 Photovoltaic properties

**Fig. 3:**
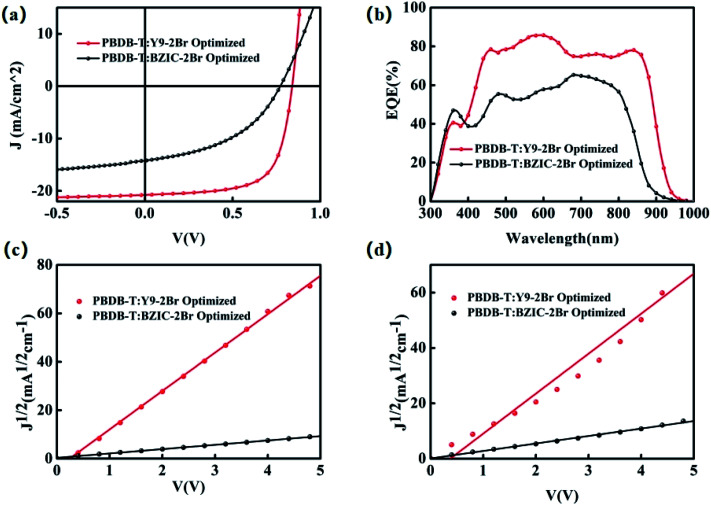
(a) The *J*–*V* curves, (b) the EQE spectra, (c) the electron mobilities and (d) the hole mobilities of the optimal OSCs based on PBDB-T:BZIC-2Br and PBDB-T:Y9-2Br, respectively.

To further investigate the photovoltaic performance, poly[(2,6-(4,8-bis(5-(2-ethylhexyl)thiophen-2-yl)-benzo[1,2-*b*:4,5-*b*′]dithiophene))-*alt*-(5,5-(1′,3′-di-2-thienyl-5′,7′-bis(2-ethyl-hexyl)benzo[1′,2′-*c*:4′,5′-*c*′]dithiophene-4,8-dione))] (PBDB-T) was chosen as the donor to match with BZIC-2Br and Y9-2Br, respectively, used as the active layer to construct OSCs, while the device structure was prepared as ITO/PEDOT:PSS/PBDB-T:BZIC-2Br(PBDB-T:Y9-2Br)/PDINO/Al (Fig. 2(c)). The current–voltage spectra measured under AM 1.5 G illumination 100 mW cm^−2^ conditions are shown in Fig. 3(a), and the corresponding parameters are listed in Table 2. The optimized OSCs based on BZIC-2Br showed a *J*_sc_ of only 15.34 mA cm^−2^ due to poor light-harvesting capability, resulting from weak absorption in the near-infrared region (NIR), and a relatively low PCE of 7.51%. However, device based on the PBDB-T:Y9-2Br (1 : 1.2 w/w) blend achieved an impressive PCE of 12.05% with a *V*_oc_ of 0.84 V, *J*_sc_ of 21.38 mA cm^−2^ and FF of 67.11%, under thermal annealing (TA) at 100 °C and the 0.5% additive 1-chloronaphthalene (CN) conditions. Moreover, Fig. 3(b) shows the EQE spectra of the PBDB-T:BZIC-2Br and PBDB-T:Y9-2Br-based optimized device. The EQE of PBDB-T:BZIC-2Br at 300–960 nm reaches 50–65%, compared with that, PBDB-T:Y9-2Br shows the wider absorption (300–990 nm) and higher EQE (75–85%), which agrees quite well with the *J*_sc_ value from the *J*–*V* curve within a 5% mismatch.

**Table 2:** The photovoltaic parameters of devices based on PBDB-T:BZIC-2Br and PBDB-T:Y9-2Br

Acceptor	D:A (w/w)	Additive, vol%	Annealing, °C	*V*_oc_, V	*J*_sc_, mA cm^−2^	FF, %	PCE, %
BZIC-2Br	1 : 1.2	CN(0.5)	100	0.75	15.34	65.19	7.51(7.21 ± 0.3)
Y9-2Br				0.84	21.38	67.11	12.05(11.75 ± 0.3)

As a result, in order to enhance the light-harvesting capability of molecules and then increase the *J*_sc_ of OSCs, it is necessary to enlarge the conjugated length of the molecules. Moreover, the effective extension of the conjugate length can elevate the LUMO energy level (shown in Fig. 2(d)) to obtain the higher *V*_oc_ and acquire the higher PCE, eventually.

### 2.4 Mobility

The space-charge limited current (SCLC) method was used to investigate the hole and electron mobilities. The dependences of the square root of current density (*J*^1/2^–*V*) on voltage are displayed in Fig. 3(c) and (d). The SCLC is described as follows.^58^
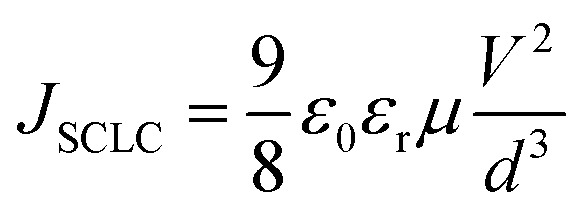
(*J* is the current density, *μ* is the hole or electron mobility, *V* is the internal voltage in the device, *ε*_r_ is the relative dielectric constant of active layer material, *ε*_0_ is the permittivity of empty space, and *d* is the thickness of the active layer.)

For the devices based-on PBDB-T:BZIC-2Br and PBDB-T:Y9-2Br, respectively, the electron mobility (*μ*_e_) were 8.90 × 10^−6^ and 2.08 × 10^−4^ cm^2^ V^−1^ s^−1^, and the hole mobility (*μ*_h_) were 6.03 × 10^−6^ and 1.86 × 10^−4^ cm^2^ V^−1^ s^−1^. The ratio (*μ*_e_/*μ*_h_) of the PBDB-T:Y9-2Br-based device (1.12) is closer to 1 than that of the PBDB-T:BZIC-2Br-based device (1.48) (as shown in Table 3), which indicates that the carrier mobility of the PBDB-T:Y9-2Br-based device is more balanced than that of the PBDB-T:BZIC-2Br-based device. The results exhibit that the effective extension of the conjugate length is a necessary measure to enhance the intermolecular charge transport and charge transfer efficiency.

**Table 3:** The electron-only mobility and hole-only mobility of devices based-on PBDB-T:BZIC-2Br and PBDB-T:Y9-2Br

	*μ*_e_ (cm^2^ V^−1^ s^−1^)	*μ*_h_ (cm^2^ V^−1^ s^−1^)	*μ*_e_/*μ*_h_
BZIC-2Br	8.90 × 10^−6^	6.03 × 10^−6^	1.48
Y9-2Br	2.08 × 10^−4^	1.86 × 10^−4^	1.12

### 2.5 Morphology

**Fig. 4:**
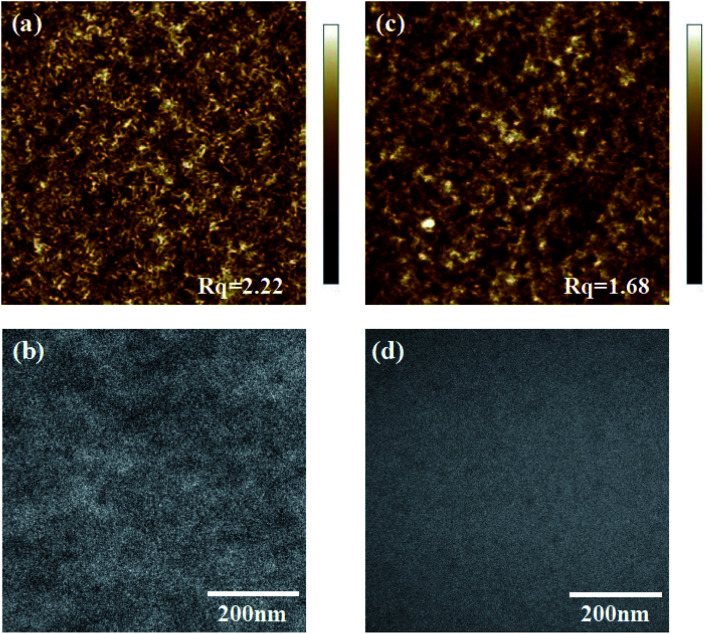
The AFM and TEM images of PBDB-T:BZIC-2Br and PBDB-T:Y9-2Br blend films. (a and b) Height image (size 5 × 5 μm^2^) of PBDB-T:BZIC-2Br and PBDB-T:Y9-2Br; (c and d) TEM image (2 × 2 μm^2^) of PBDB-T:BZIC-2Br and PBDB-T:Y9-2Br.

The morphology of OSCs plays a key role in photovoltaic properties. Good miscibility is desirable for maximum exciton separation and transport. To evaluate the effects of the microstructure of active layers on device performance, the morphologies of PBDB-T:BZIC-2Br and PBDB-T:Y9-2Br blend films were investigated by atomic force microscopy (AFM). As is shown in Fig. 4(a) and (c), the root-mean-square (*R*_q_) surface roughness value for the blend films of PBDB-T:BZIC-2Br and PBDB-T:Y9-2Br are 2.22 nm and 1.68 nm, respectively. The blend film of PBDB-T:Y9-2Br exhibits the smooth morphology with smaller *R*_q_, which can also is observed in TEM (Fig. 4(b) and (d)). These morphological features may lead to the remarkably high *J*_sc_ and FF of the devices.

## 3. Conclusions

In summary, two non-fullerene acceptors using the A–DA′D–A structure multifused benzotriazole central core are designed and synthesized. Compared with BZIC, the absorption spectrum of BZIC-2Br exhibits red-shift due to the narrower optical bandgap. By using a medium bandgap polymer PBDB-T as the electron donor, OSCs based on PBDB-T:BZIC-2Br and PBDB-T:Y9-2Br exhibit different properties. Compared with BZIC-2Br, PBDB-T:Y9-2Br exhibits the more outstanding performance. The obvious red-shift (about 51 nm) of the absorption spectrum and LUMO upshift of Y9-2Br in the thin film can simultaneously increase *V*_oc_ and *J*_sc_, and good morphological can acquire high FF. The results demonstrate that the introduction of bromine can enhance the light-harvesting capability and reduce the bandgap, and effective extension of the conjugate length can regulate the HOMO and LUMO energy levels to decrease energy levels, enhance the light-harvesting capability of molecules, increase the absorption and reduce the bandgap and enhance charge mobility. These changes will in turn contribute to the remarkably high *V*_oc_ of 0.84 V, *J*_sc_ of 21.38 mA cm^−2^ and an FF of 67.11%, and PCE of 12.05% achieved by the devices.

## Conflicts of interest

There are no conflicts to declare.

## Supplementary Material

RA-011-D1RA01348C-s001
