# Supplementary material for: Bromination and increasing the molecular conjugation length of the non-fullerene small-molecule acceptor based on benzotriazole for efficient organic photovoltaics
Source: RSC Adv. 2021 Apr 13;11(22):13571–8. doi: 10.1039/d1ra01348c (PMC8697487; doi:10.1039/d1ra01348c)
Supplement: RA-011-D1RA01348C-s001 [file RA-011-D1RA01348C-s001.pdf]

Electronic Supplementary Material (ESI) for RSC Advances.
This journal is © The Royal Society of Chemistry 2021

Supporting Information

Bromination and Increasing the Molecular Conjugation Length of the Non-fullerene Small-Molecule Acceptor Based on Benzotriazole for Efficient Organic Photovoltaics

Na Zhang, Zhe Li, Can Zhu, Hongjian Peng*,Yingping Zou

College of Chemistry and Chemical Engineering, Central South University, Changsha, Hunan 410083, China

Corresponding author：Hongjian Peng; Tel: +86−731−88879616; E-mail: hongjianpeng@126.com

EXPERIMENTAL SECTION

1. Characterization of information

2. Fabrication of devices

3. Hole mobility and electron mobility measurements

4. Mass spectrum and thermogravimetric analysis

5. Synthesis

6. The figures of ^{1}H NMR

1. Characterization of information

^{1}H Nuclear Magnetic Resonance (^{1}H NMR) was recorded using Bruker DMX-400 spectrometer with deuterated chloroform as solvent at 293 K. Chemical shifts were reported as δ values (ppm) with tetramethylsilane (TMS) and$CHCl_3$ (Chemical shift $\delta = 7.26$ ppm for ^{1}H NMR) as the internal references. UV-Vis absorption spectra were recorded on the Shimadzu UV-2600 spectrophotometer. Cyclic voltammetry (CV) was recorded with a computer controlled CHI 660E electrochemical workstation using BZIC-2Br (Y9-2Br) film on platinum electrode (1.0 cm^2) as the working electrode, a platinum wire as the counter electrode and Ag/AgCl (0.1 M) was used as reference electrode in an anhydrous and argon-saturated solution of 0.1 M of tetrabutylammonium hexafluorophosphate (Bu_4NPF_6) in acetonitrile at a scan rate of 20 mV s^{-1}. The morphologies of the PBDB-T:BZIC-2Br(PBDB-T:Y9-2Br)blend films were investigated by atomic force microscopy (AFM, Agilent Technologies, 5500 AFM/SPM System, USA) in contacting mode with a 5 μm scanner. Transmission electron microscope (TEM) measurements were performed in a JEM-2100F.

2. Fabrication of devices

Solar cells were fabricated in the configuration of the traditional structure with an indium tin oxide (ITO) glass positive electrode and a metal Al negative electrode. The ITO glass was cleaned by sequential ultrasonic treatment in detergent, deionized water, acetone and isopropanol, and UV-treated in ultraviolet-ozone chamber (Jelight Company, USA) for 20 min. The PEDOT:PSS (poly(3,4-ethylene dioxythiophene): poly(styrene sulfonate)) (Baytron PVP Al 4083, Germany) was filtered through a 0.45 μm filter and spin coated at 2500 rpm for 50 s on the ITO substrate. The PBDB-T:BZIC-2Br (PBDB-T:Y9-2Br) (D:A = 1:1.2, ω/ω; 16 mgmL^{-1} in total) were dissolved in chloroform (CF) and spin-cast at 3500 rpm for 30 s onto the PEDOT:PSS layer. It was then annealed at 100°Cfor 5 min. After cooling to room temperature, methanol solution of PDINO at a concentration of 1.0 mgmL^{-1} was deposited atop the active layer at 2500 rpm for 50 s to afford a thickness of 15 nm. Finally, top Al electrode was deposited in vacuum onto the cathode buffer layer at a pressure of

1×10^{-5} Pa. The active area of the device was 5 mm^2.

The *J*−*V* measurement was performed via the solar simulator (SS-F5-3A, Enlitech) along with AM 1.5 G spectra whose intensity was calibrated by the certified standard silicon solar cell (SRC-2020, Enlitech) at 100 $mWcm^{-2}$. The external quantum efficiency (EQE) values were measured by Solar Cell Spectral Response Measurement System QE-R3-011 (Enli Technology Co., Ltd., Taiwan). The light intensity at each wavelength was calibrated with a standard single-crystal Si photovoltaic cell.

3. Hole mobility and electron mobility measurements

The device structures of hole (μ_h) and electron (μ_e) mobilities are ITO/PEDOT:PSS/active layer/MoO_3/Ag and ITO/ZnO/active layer/PDINO/Al, respectively. The hole and electron mobilities are calculated according to the space charge limited current (SCLC) equation: $J = 9\mu\varepsilon_r\varepsilon_0V^2 /8d^3$, where J is the current density, μ is the hole or electron mobility, V is the internal voltage in the device, ε_r is the relative dielectric constant of active layer material, ε_0 is the permittivity of empty space, and d is the thickness of the active layer.

4. Mass spectrum and thermogravimetric analysis

The molecular mass was confirmed by using an Autoflex III matrix-assisted Laser desorption ionization mass spectrometer (MALDI-TOF-MS). Thermogravimetric analysis (TGA) was conducted on a Perkin-Elmer TGA-7 with a heating rate of 10 K min^{-1} under nitrogen.

5.Synthesis

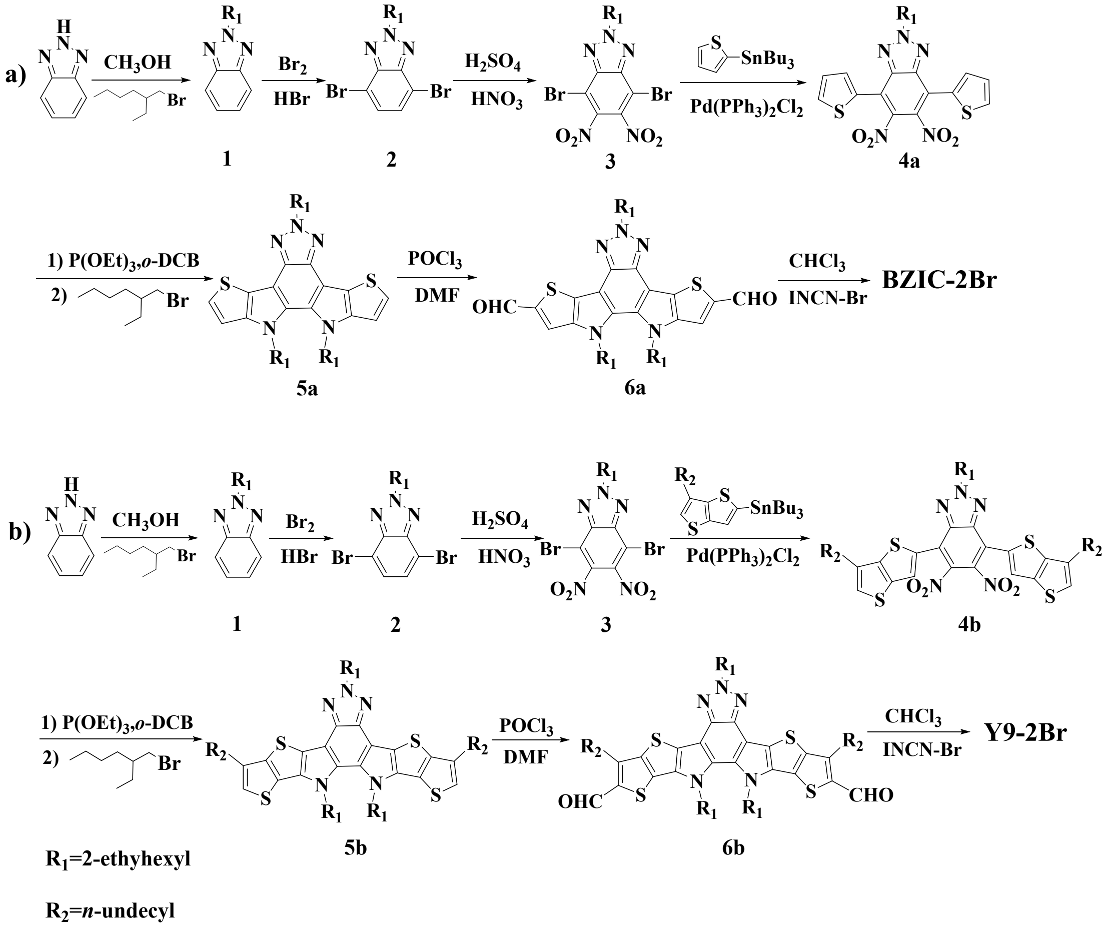

Scheme 1. The synthetic routes of BZIC-2Br and Y9-2Br.

5.1 Detailed procedures

Compound 1:

Benzotriazole(15.540g), potassium tert-butoxide(15.380g), tetrabutylammonium bromide(0.5g) were dissolved in methyl alcohol(60-80mL) with increasing speed of dissolution by heating and then above solution was added to the 500mL three-necked flask. After cooling, above system which has added bromo-iso-octane reacted 24 h under 90℃ reflux condensation. Solution reacting was completely cooled down to ambient temperature and quenched with water. Finally, the reaction mixture was extracted with dichloromethane (DCM),the organic layer was removed by a rotary evaporator and the residue was purified by silica gel column only using petroleum

ether (PE) to get a kind of light yellow oily liquid, **compound1**(15.451g,51.2%).

^{1}H NMR (400 MHz,$CDCl_3$) δ 7.87 (d,J = 8.4 Hz,2H),7.36 (d,J = 6.7 Hz,2H), 4.63 (d,J = 7.1 Hz,2H),2.24 (dt,J=12.0,5.9 Hz,1H),1.36-1.26 (m,7H),0.96-0.85 (m,7H).

Compound 2:

Compound1 (15.451g)having been compounded and HBr(80 mL) were added to the 500mL three-necked flask and then reacted 1 h under 100℃.Next,Br_2(9.42mL) were dropwise added to system by constant pressure drip funnel meanwhile reacted with stirring 10 h under 100℃.When reaction completed, pH of system that had been cooled down to ambient temperature was adjusted by $NaCO_3$(or NaOH) till pH showed neutral. Finally, the reaction mixture was extracted with DCM, the organic layer was removed by a rotary evaporator and the residue was purified by silica gel column only using PE to get **compound2**(17.907 g,68.9 %).

^{1}H NMR (400 MHz,$CDCl_3$) δ 7.10 (s,1H),4.44 (d,J = 7.1 Hz,1H), 2.05-1.99 (m,1H),1.34-1.26 (m, 7H),0.94-0.84 (m, 7H).

Compound 3:

In ice-water bath circumstance, concentrated nitric acid(HNO_3)(57.982 g) were dropwise added to the 500mL three-necked flask with concentrated sulphuric acid(H_2SO_4)(98%,360.781 g).**Compound2** were dropwise added to system under 50℃ and mixture reacted with stirring 4 h. When reaction completed, solution was transferred to 1000mL beaker with ice-water. And then, the reaction mixture was extracted with dichloromethane (DCM),the organic layer was removed by a rotary evaporator and the residue was purified by silica gel column using DCM/PE (1/3, v/v) to get**compound3**(19.601 g，88.9 %).

Compound 4a and **4b:**

Compound4a has the same equivalent as **compound4b**.

Tributyl(thiophen-2-yl)stannane(45.801 g) and **compound3**(19.601 g) were dissolved in tetrahydrofuran(THF) under argon atmosphere that kept 15 min. And the catalyst, $Pd(PPh_3)_2Cl_2$ (1.750 g), was added to system, and mixture reacted with stirring 48 h at 70℃. Solution reacting completely was cooled down to ambient temperature and quenched with water meanwhile THF were removed by a rotary evaporator. Finally, the reaction mixture was extracted with dichloromethane (DCM),the organic layer was removed by a rotary evaporator and the residue was purified by silica gel column using DCM/PE (1/3, v/v) to get **compound4a**(18.375 g,92.5 %)(**compound 4b:**4.06 g, 81.2%).

compound 4a: ^1H NMR (400 MHz,$CDCl_3$) δ 7.66 (d, J = 5.0 Hz, 2H), 7.55 (d, J = 3.5 Hz, 2H), 7.23-7.19 (m, 2H), 4.75 (d, J = 6.8 Hz, 2H), 2.25 (dd, J = 12.2, 6.1 Hz, 1H)
compound 4b: ^1H NMR (400 MHz, $CDCl_3$) δ 7.22 (d, *J* = 8.1 Hz, 2H), 6.94 (s, 2H), 2.71 (dd, *J* = 16.5, 8.7 Hz, 6H), 1.39-1.22 (m, 44H), 0.95-0.89 (m, 12H).

Compound 5a and **5b:**

Compound5a has the same equivalent as **compound5b**.

Compound4b (8.577 g, 9.46 mmol)) and triethyl phosphate (7.8 g, 47.3mmol)were dissolved in the *o*-dichlorobenzene (*o*-DCB, 100 mL) under nitrogen. After being heated at 180℃ for 12 h, the aqueous phase was extracted with ethylacetate and the organic layer was dried over Na_2SO_4. The solvent was removed under vacuum. Crude product was obtained as a dark green liquid without further purification.

Crude product, 1-bromo-2-ethylhexane (16.6 g, 86.25 mmol), potassium iodide (0.6 g, 3.8 mmol) and potassium carbonate (5.26 g, 94.6 mmol) were dissolved in the N,N-dimethylmethanamide (DMF, 60 mL). After being heated at 90℃ overnight, the solution was removed under vacuum and extracted with ethylacetate and H_2O. The organic layers were combined and dried over MgSO4, filtered and purified with column chromatography on silica gel using DCM/PE (1/5, v/v) as the eluent to give a light-yellow solid, **compound5b** (1.5 g, 60.4% yield)(**compound5a:** 5.476 g，64.9

%).

compound 5a:^{1}H NMR (400 MHz, $CDCl_3$)δ7.27 (d, J = 5.1 Hz, 2H), 7.06 (d, J = 5.1 Hz, 2H), 4.64 (d, J = 7.1 Hz, 2H), 4.39 (d, J = 7.6 Hz, 4H), 2.34-2.23 (m, 1H), 1.87-1.80 (m, 2H), 1.23-1.17 (m, 9H), 0.88 (t, J = 6.8 Hz, 9H), 0.81-0.68 (m, 24H).

compound 5b:^{1}H NMR (400 MHz, $CDCl_3$) δ 6.98 (s, 2H), 4.72 (d, J = 7.2 Hz, 2H), 4.58 (d, J = 7.7 Hz, 4H), 2.81 (t, J = 7.7 Hz, 4H), 1.46-1.23 (m, 50H), 1.01-0.75 (m, 30H), 0.58 (dd, J = 13.2, 7.4 Hz, 7H).

Compound 6a and **6b:**

Compound6a has the same equivalent as **compound6b**.

To a solution of **compound5b**(1.5 g, 1.47 mmol) in DMF (50 mL) at 0°C was added phosphorus oxychloride (2.1 ml, 22.05 mmol) dropwise slowly under nitrogen. The mixture was stirred at 0°C for 2 h, and then the solution was heated to 90℃ and stirred overnight. The reaction mixture was poured to ice water (100 mL), neutralized with saturated sodium hydroxide solution, and then extracted with dichloromethane twice. The combined organic layer was washed with water and brine, dried over $MgSO_4$, and evaporated under reduced pressure. The crude product was purified by column chromatography using DCM / PE (1/1, v/v) to obtain **compound6b** (540 mg, 62.3%) as a yellow solid. (**compound6a:**4.745 g，79.7 %)

compound 6a: ^{1}H NMR (400 MHz，$CDCl_3$) δ 9.92 (s，2H)，7.77 (s，2H)，4.59 (d，J = 7.0 Hz，2H)，4.48 (d，J = 7.6 Hz，4H)，2.24-2.16 (m，1H)，1.86 (dd，J = 12.0，6.0 Hz，2H)，0.78 – 0.73 (m，30H)，0.61-0.54 (m，6H)，0.50-0.45 (m，6H).

compound 6b: ^{1}H NMR (400 MHz, $CDCl_3$) δ 10.12 (s, 2H), 4.74-4.59 (m, 6H), 3.19 (t, *J*=7.7 Hz,3H), 1.95-1.90 (m, 4H), 1.46-1.24 (m, 44H), 0.91 (dddd, *J* = 22.2, 17.4, 12.8, 4.9 Hz, 28H), 0.59 (ddd, *J*=37.5, 15.8, 7.2 Hz, 12H).

CompoundBZIC-2Br:

Compound6a(0.137g, 0.195 mmol) and terminal group(0.174 g, 0.602 mmol) , pyridine (1 mL) and chloroform (30 mL) were dissolved in a round bottom flask

under nitrogen. Then the mixture was stirred and refluxed overnight. After removing the solvent, the crude product was purified with column chromatography on silica gel using DCM / PE (1/2, v/v) to afford 162 mg of **compound BZIC-2Br** in 66.7% yield as a dark blue solid.

^{1}H NMR (400 MHz, $CDCl_3$) δ9.02 (s，2H)，8.84 (s，2H)，8.56 (d，J = 8.4 Hz，2H)，8.05 (d，J = 9.9 Hz，2H)，7.85 (d，J = 21.0 Hz，2H)，4.75-4.68 (m，2H)，4.53 (d，J = 7.6 Hz，4H)，2.31 (s，1H)，1.89 (s，2H)，1.40-1.31 (m，15H)，1.03-0.80(m，18H)，0.65 (dt，J = 26.7 Hz，9H).

CompoundY9-2Br:

Compound6b(0.138g, 0.12 mmol) and terminal group(0.350g, 1.21 mmol) , pyridine (1 mL) and chloroform (30 mL) were dissolved in a round bottom flask under nitrogen. Then the mixture was stirred and refluxed overnight. After removing the solvent, the crude product was purified with column chromatography on silica gel using DCM / PE (1/1, v/v) to afford 100 mg of **compound Y9-2Br** in 50% yield as a dark blue solid.

^{1}H NMR (400 MHz, $CDCl_3$) δ 9.17 (s, 2H), 8.70 (dd, J = 5.7, 2.5 Hz, 2H), 7.95 (dd, J = 5.3, 3.1 Hz, 2H), 7.86-7.66 (m, 4H), 4.73 (dd, J = 15.0, 7.4 Hz, 6H), 3.23 (t, J = 7.7 Hz, 3H), 1.96-1.81 (m, 4H), 1.56-1.20 (m, 44H), 1.16-0.56 (m, 40H).

HRMS (MALDI-TOF-MS):*m/z* Calcd for $C_{90}H_{106}Br_2N_9O_2S_4$ $[M + H]^+$1630.5719, found 1630.5662.

6. The figures of ^{1}H NMR, mass spectrum and thermogravimetric analysis

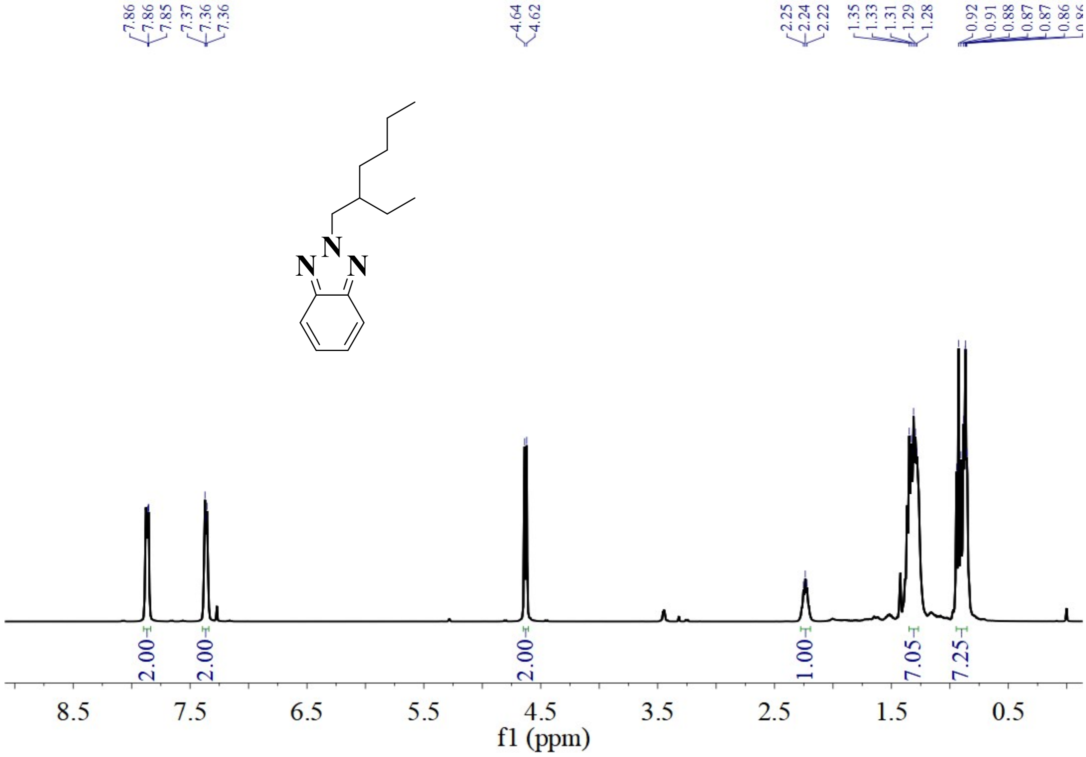

Fig. S1 ^{1}H NMR spectrum of**compound1**

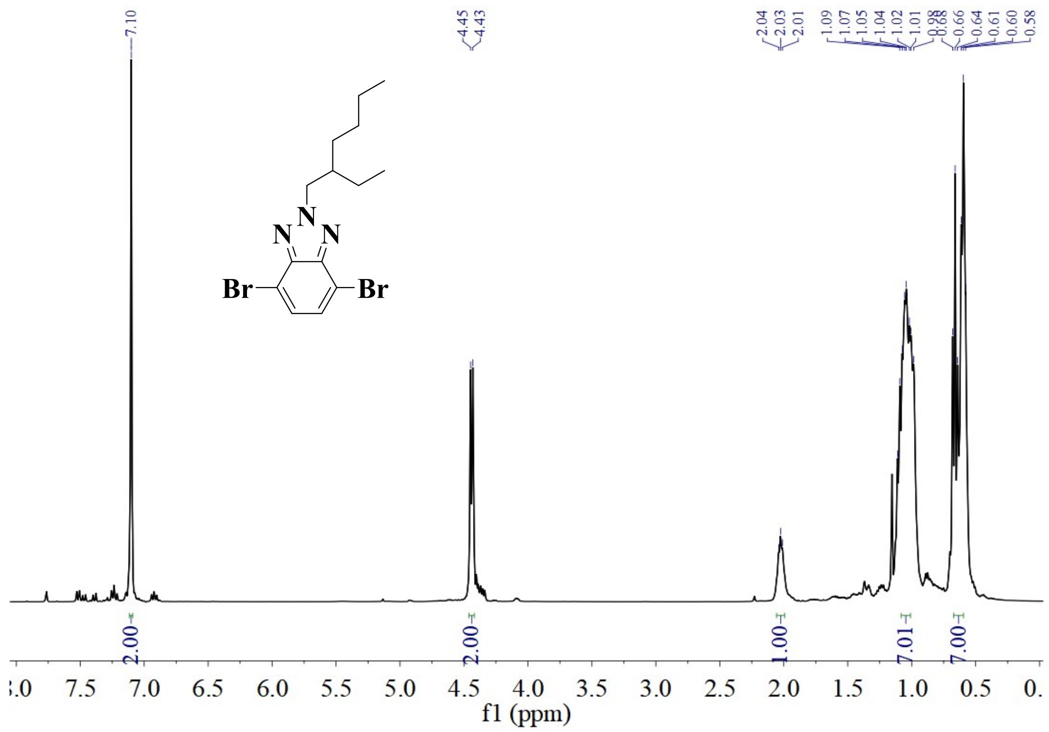

Fig. S2 ^{1}H NMR spectrum of **compound 2**

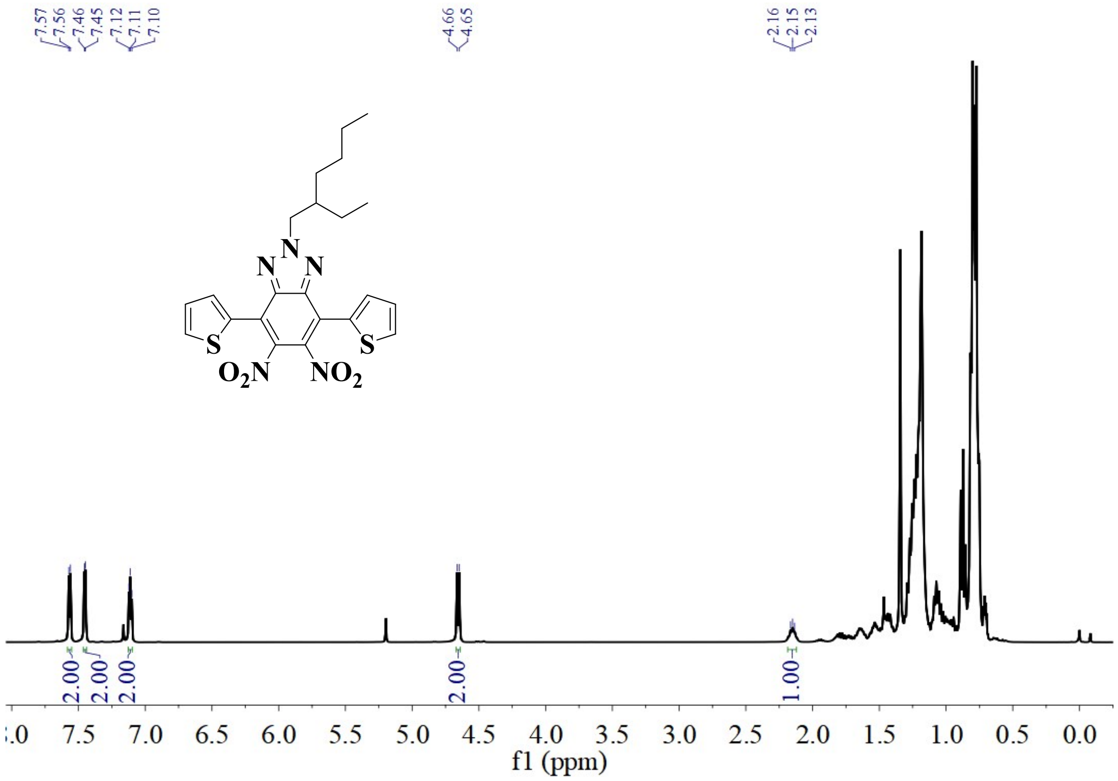

Fig. S3 ^{1}H NMR spectrum of **compound 4a**

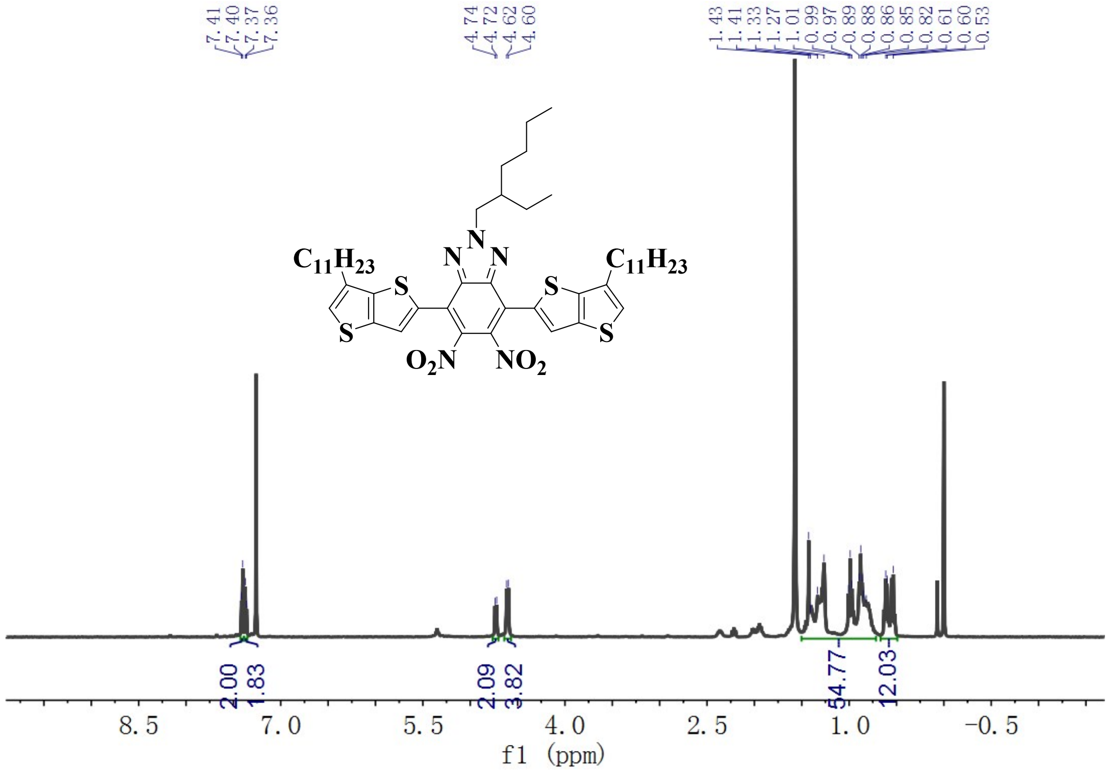

Fig. S4 ^{1}H NMR spectrum of **compound 4b**

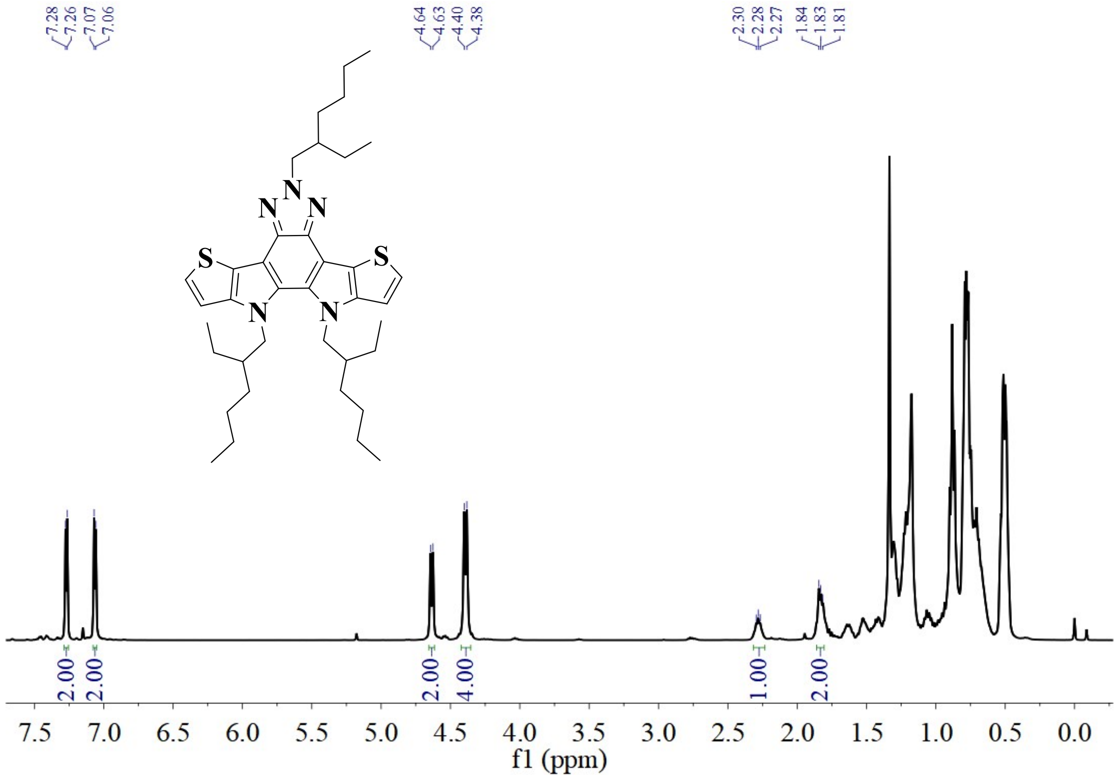

Fig.S5 ^{1}H NMR spectrum of **compound 5a**

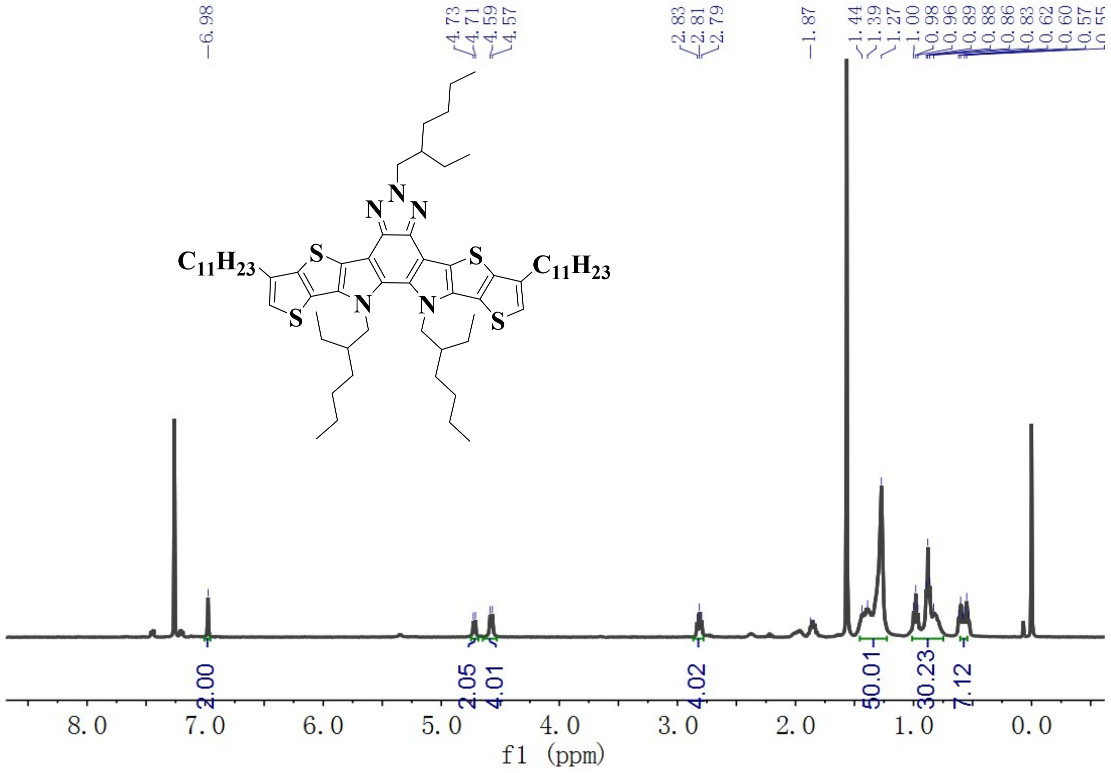

Fig.S6 ^{1}H NMR spectrum of **compound 5b**

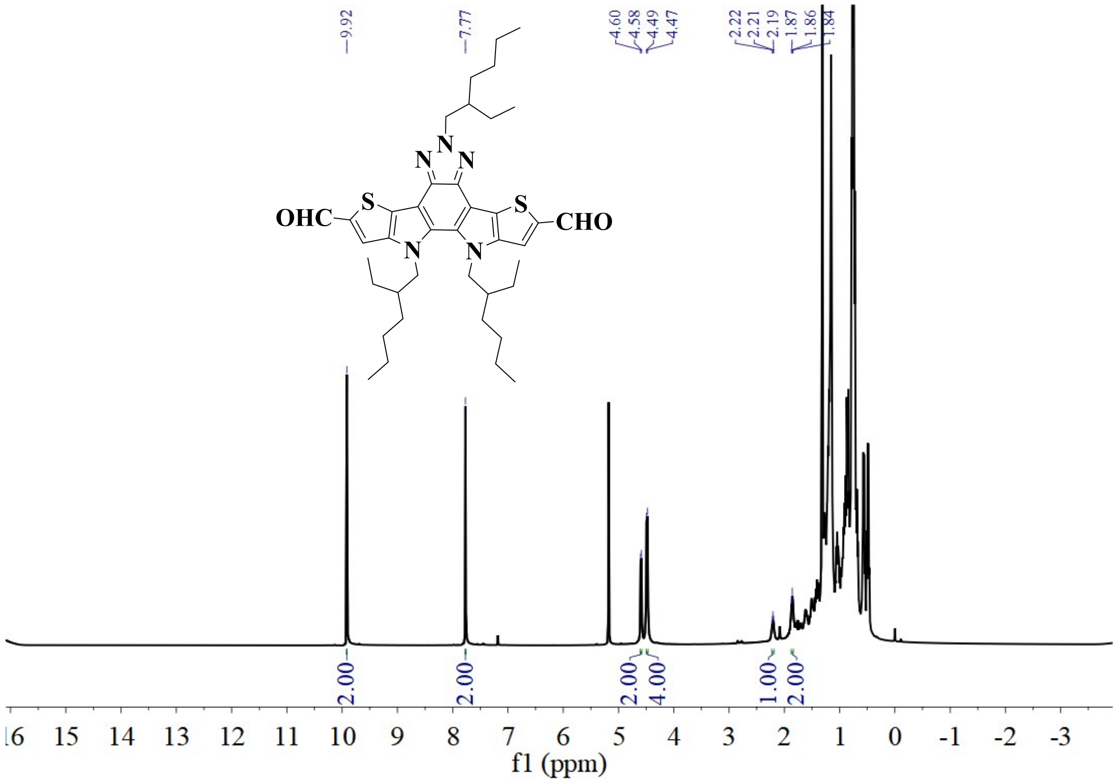

Fig. S7 ^{1}H NMR spectrum of**compound 6a**

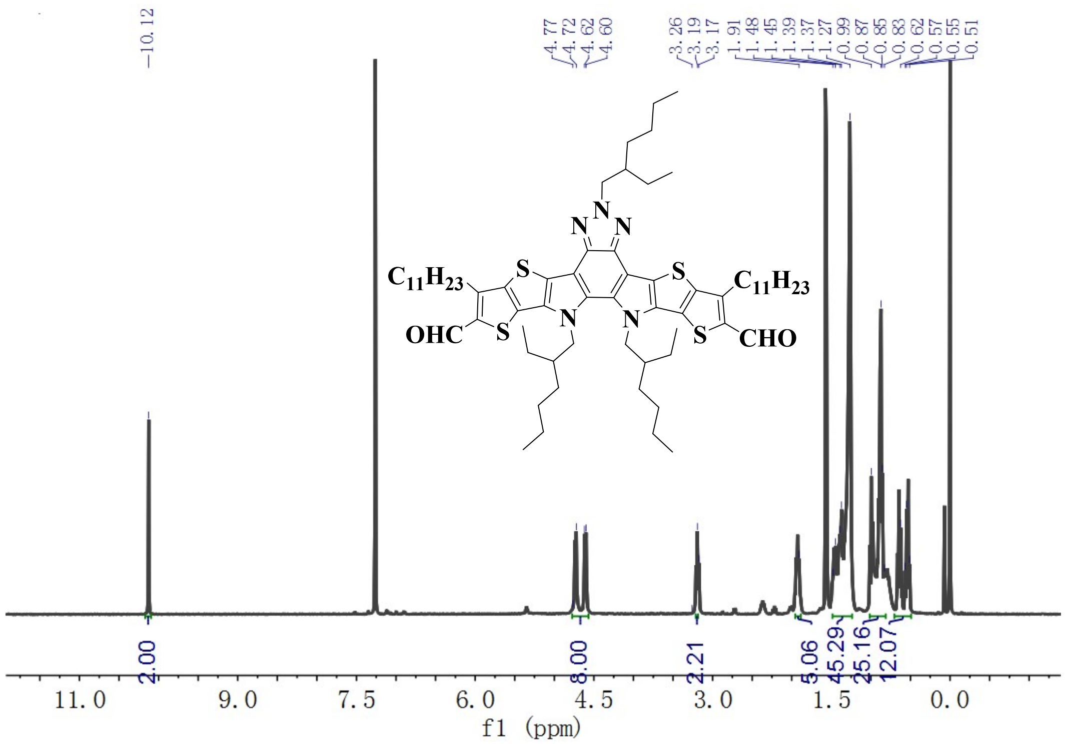

Fig. S8 ^{1}H NMR spectrum of **compound 6b**

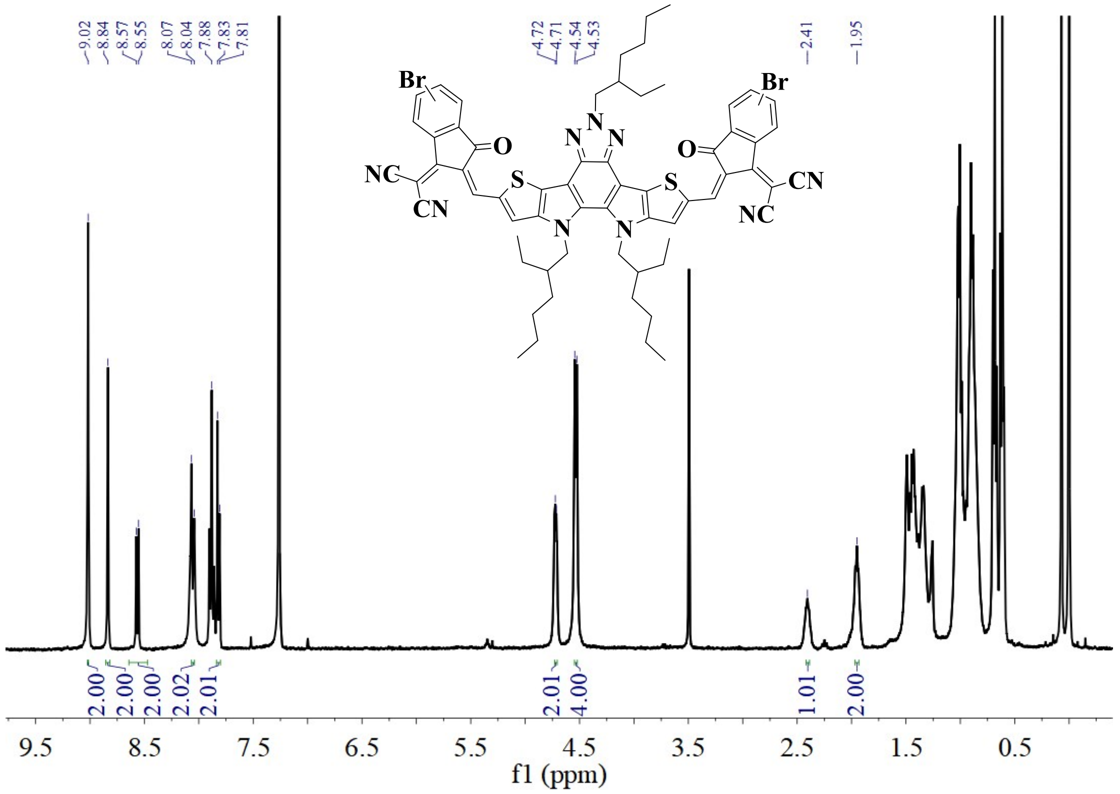

Fig. S9 ^{1}H NMR spectrum of **compound BZIC-2Br**

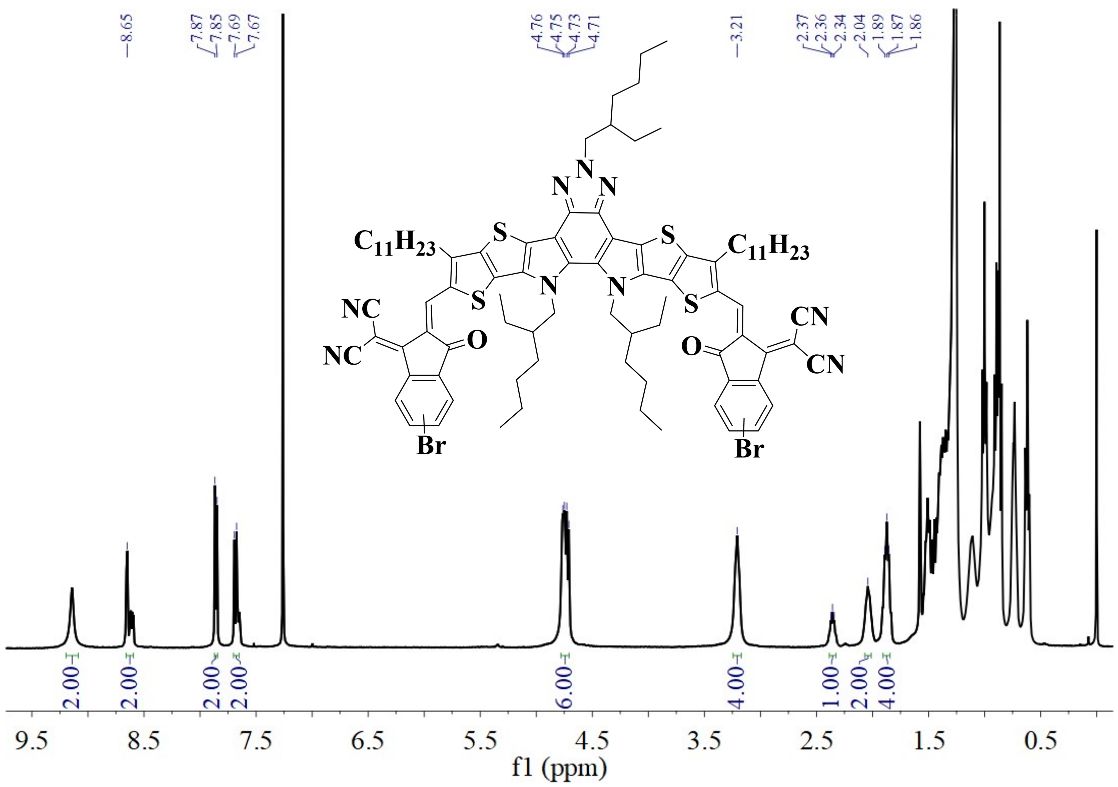

Fig. S10 ^{1}H NMR spectrum of **compound Y9-2Br**

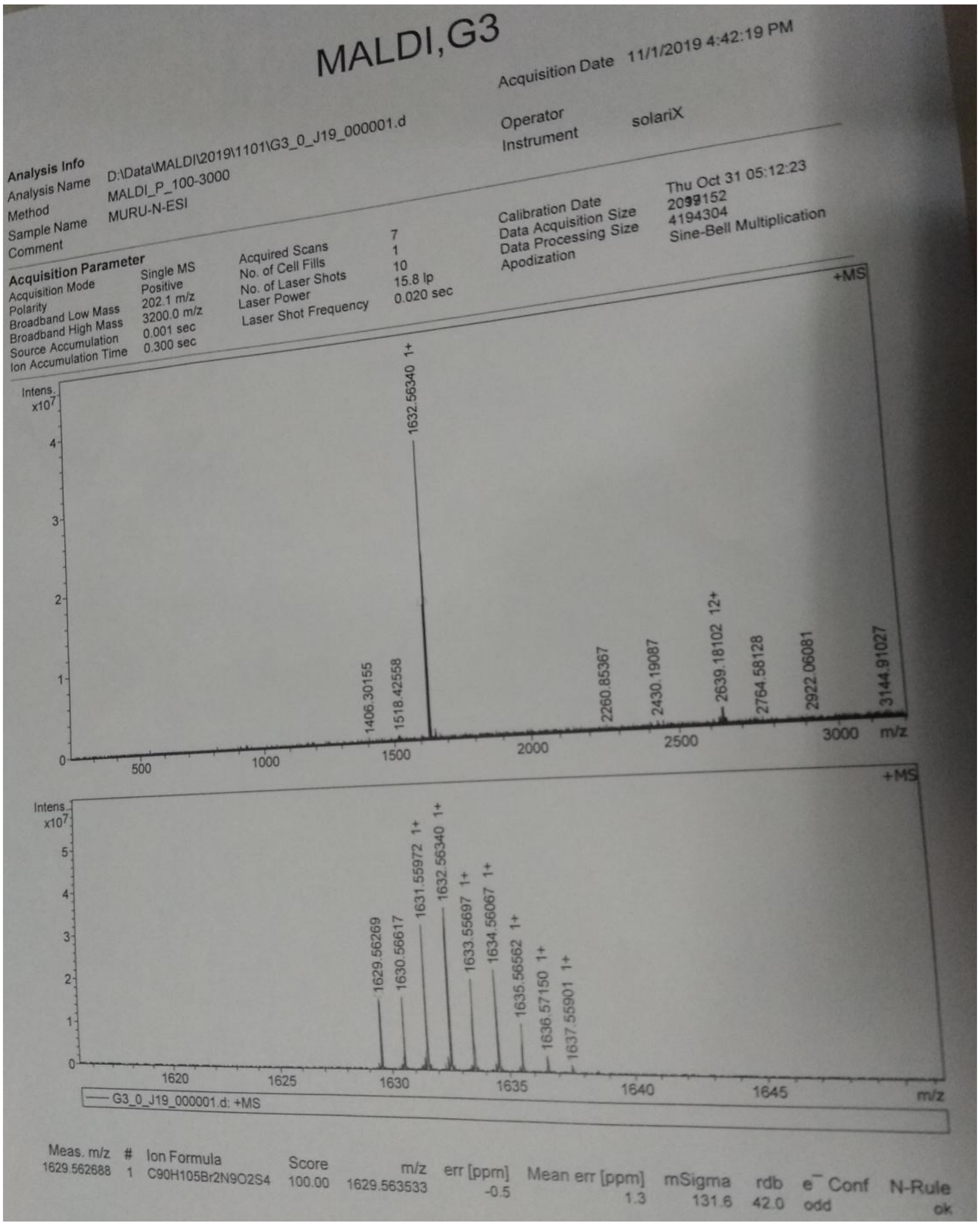

Fig. S11 The high resolution mass spectrum (MALDI-TOF-MS) of **compound Y9-2Br**

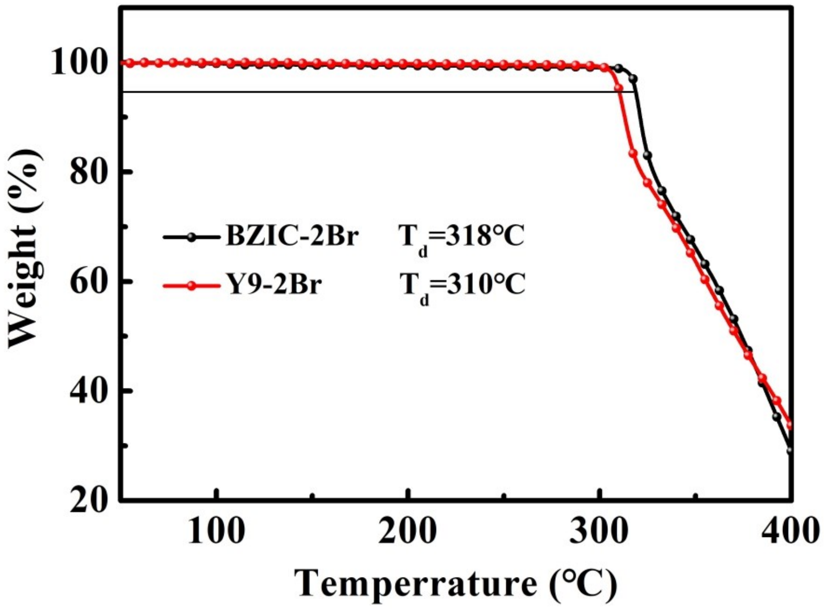

Fig. S12 Thermogravimetric analysis curve of BZIC-Br and Y9-2Br with heating rate of 10℃ min^{-1}
